# Lack of Effects of Metformin and AICAR Chronic Infusion on the Development of Hypertension in Dahl Salt-Sensitive Rats

**DOI:** 10.3389/fphys.2017.00227

**Published:** 2017-04-20

**Authors:** Tengis S. Pavlov, Vladislav Levchenko, Daria V. Ilatovskaya, Hui Li, Oleg Palygin, Nuria M. Pastor-Soler, Kenneth R. Hallows, Alexander Staruschenko

**Affiliations:** ^1^Department of Physiology, Medical College of WisconsinMilwaukee, WI, USA; ^2^Division of Hypertension and Vascular Research, Henry Ford HospitalDetroit, MI, USA; ^3^Division of Nephrology and Hypertension, Department of Medicine, Keck School of Medicine, University of Southern California, Los AngelesLos Angeles, CA, USA

**Keywords:** hypertension, salt sensitivity, AMPK, metformin, AICAR, ENaC, blood pressure

## Abstract

In the kidney, reabsorption via the epithelial sodium channel (ENaC) is involved in long-term blood pressure control. Previously we demonstrated that ENaC hyperactivity is associated with development of salt-sensitive (SS) hypertension in Dahl SS rats. AMP-activated kinase (AMPK), playing a role in cellular energy homeostasis, has been shown to decrease ENaC activity. Here, we tested whether metformin and AICAR, two drugs that activate AMPK, affect the development of salt-induced hypertension. High salt diet significantly increased mean arterial pressure (MAP) in Dahl SS rats. Blood pressure elevation was accompanied by a short-term decline of heart rate and increased circadian arterial pressure dipping. Metformin and AICAR were delivered intravenously at doses of 200 and 20 mg/kg/day, respectively. However, both control and drug-treated groups had similar development of high blood pressure within 3 weeks of 8% NaCl dietary salt intake. In the metformin-treated animals MAP reached 164.9 ± 9.1 mmHg, which was not significantly different from the control group (171.8 ± 5.6 mmHg). Patch clamp analysis revealed that the metformin-treated rats had no difference in the activity of ENaC. AICAR treatment also did not affect the development of hypertension and kidney injury. MAP reached 182.8 ± 4.8 and 178.0 ± 2.8 mmHg in AICAR and vehicle treated groups, respectively. Of note, we found that high-salt diet activated AMPK in the Dahl SS rats, and treatment with these AMPK activators had no significant further effect on AMPK activity. We conclude that AMPK activators, at least under these conditions, do not affect development of hypertension during high-salt diet in the Dahl SS rat model.

## Introduction

Activators of 5′-AMP-activated protein kinase (AMPK) are widely used for treatment of metabolic disorders, type 2 diabetes, heart disease, anti-proliferative therapy and sports medicine. AMPK, a heterotrimeric serine/threonine protein kinase, is activated in response to reduced intracellular ATP to AMP ratio and controls numerous metabolic pathways. Activation of this kinase improves glucose uptake, fatty acid oxidation and insulin sensitivity in skeletal muscles, carbohydrate and lipid metabolism in liver and adipocytes, and release of insulin by pancreatic β cells (Long and Zierath, 2006).

A variety of medications work through AMPK and its downstream pathways. For instance, the biguanide drug metformin, in use since the 1950s, is a very effective agent for the treatment of type 2 diabetes, insulin resistant states, pre-diabetes, and cancer (Rojas and Gomes, 2013). Metformin acts primarily in the liver by reducing glucose output and, secondarily, by augmenting skeletal myocyte glucose uptake. Many mechanisms are involved in the beneficial effects of metformin on cellular metabolism; the activation of an upstream kinase, liver kinase B1 (LKB1), increased cellular AMP levels, and consequent AMPK activation are considered to be key mechanisms for the action of this drug (Zhou et al., 2001; Grahame Hardie, 2014).

Another example of AMPK axis targeting medications is AICAR (5-amino-4-imidazole carboxamide ribonucleoside), an AMP-mimetic and AMPK-regulating agent that is currently being investigated for the treatment of metabolic syndrome and as an ischemic preconditioning agent. Intravenous AICAR administration reduces hepatic glucose output, inhibits whole body lipolysis and lowers blood glucose concentrations in type 2 diabetic patients (Boon et al., 2008). Moreover, AICAR has a prominent effect on skeletal muscles, enhancing glucose uptake, training adaptation, and increasing endurance (Narkar et al., 2008). Finally, pre- and peri-operative AICAR infusion in patients undergoing coronary artery bypass graft surgery reduces early cardiac death, myocardial infarction, and combined adverse cardiovascular outcomes (Mangano, 1997).

Altered sodium metabolism is a consistent finding in diabetes, and it is present even in the absence of overt renal or cardiac disease. Both insulin-dependent and non-insulin-dependent diabetic individuals have been reported to have a significant increase of total exchangeable sodium relative to non-diabetic controls (O'hare et al., 1985; Bickel et al., 2002). In cultured cells, AMPK was shown to regulate renal epithelial currents mediated by ENaC (Bhalla et al., 2006), ROMK (Siraskar et al., 2013), and CFTR (Takiar et al., 2011). Analysis of renal function in mice with genetic deletion of the AMPK α_1_ catalytic subunit showed normal renal sodium handling and a moderate antidiuretic state that might indicate a compensatory upregulation in α_2_ subunit. A renal epithelium-specific knock down of α_2_ subunit in the α_1_ knockout strain was accompanied by reduced renal AMPK activity and a moderate salt/water wasting phenotype on normal and high salt diets (Lazo-Fernández et al., 2017).

The Dahl salt-sensitive rat strain is a well-established model for studies of the NaCl-dependent type of hypertension. This model recapitulates many traits of the human form of salt-sensitive hypertension: suppressed plasma renin activity, lower circulating aldosterone concentration, increased vascular resistance, impaired pressure-natriuresis response, and decreased venous compliance (Rapp, 1982; Rapp et al., 1989; Sullivan, 1991; Cowley, 1997; Hall, 2016). A hallmark of Dahl SS rats is the rapid progression of hypertension in response to high-NaCl diet. Typical experimental protocols utilizing 4 or 8% NaCl chow induce a substantial rise in blood pressure (BP) within 2–3 weeks after the change of diet.

Sodium reabsorption via ENaC in the aldosterone-sensitive distal nephron (ASDN) is one of the major factors that regulate natriuresis and water-electrolyte balance in the organism. In pathological conditions, increased ENaC activity is associated with improper sodium handling, edema, excessive fluid retention, and hypertension. For instance, hypertension is typical for patients with Liddle's syndrome caused by gain-of-function mutations in ENaC (Hansson et al., 1995). We and others have shown that in Dahl SS rats abnormal activation of ENaC on a high salt diet contributes to the development of hypertension (Kakizoe et al., 2009; Pavlov et al., 2013; Pavlov and Staruschenko, 2016).

It is currently unclear whether AMPK activators may be beneficial in the treatment of salt-sensitive hypertension. Experiments on airway tissue cultures reveal that ENaC-dependent short-circuit currents are substantially inhibited by AMPK activation (Woollhead et al., 2007; Myerburg et al., 2010). Zhang and colleagues did not find any effects of metformin on salt-induced hypertension in Dahl SS rats (Zhang et al., 1994). However, in later studies intracerebroventricular administration of metformin was found to prevent salt-induced blood pressure increase in the spontaneously hypertensive rat (SHR) model (Petersen et al., 2001). Recently, Yu et al. demonstrated that chronic administration of caffeine augments AMPK activity in Dahl SS rats and attenuates hypertension via a decrease of ENaC activity in the ASDN and improved natriuresis (Yu et al., 2016). In the current study, we used chronic intravenous metformin and AICAR treatments to test how these AMPK activators affect the development of SS hypertension and ENaC activity in Dahl SS rats.

## Methods

### Animals and surgery

**Figure 1:**
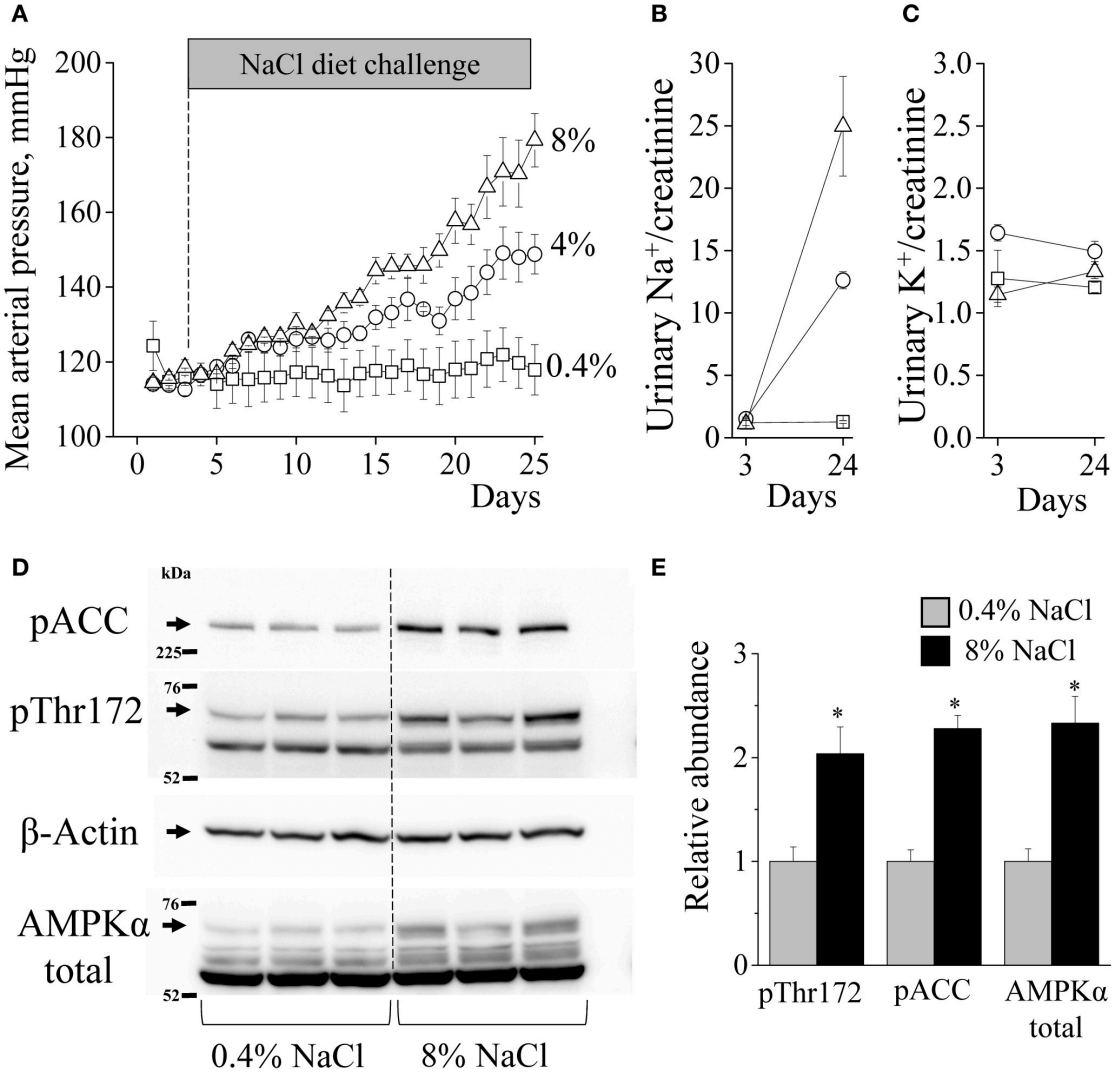
**AMPK activity is increased in salt-induced hypertension. (A)** Mean arterial blood pressure in Dahl SS rats fed a 0.4% (*n* = 4), 4% (*n* = 3), or 8% (*n* = 5) NaCl diet measured with DSI telemetry. Sodium **(B)** and potassium **(C)** urinary excretion (mM/mg of creatinine) before and after diet challenge (*n* = 5 in each group). **(D)** Western blot of kidney cortex lysates isolated from the animals on 0.4 and 8% diets. Samples were tested for expression of phosphorylated acetyl-CoA carboxylase (pACC) and AMPKα levels as indicators of AMPK pathway activity. **(E)** Summary graph of pThr172-AMPKα, p-ACC and AMPKα abundances, along with β-actin as a loading control (^*^p < 0.05 vs. 0.4% NaCl for each parameter, unpaired *t*-tests, *n* = 3).

Experiments were performed on Rapp Dahl salt-sensitive (SS) rats (SS/JrHsdMcwi) bred at the Medical College of Wisconsin. Animal use and welfare procedures adhered to the National Institutes of Health Guide for the Care and Use of Laboratory Animals following protocols reviewed and approved by the Medical College of Wisconsin Institutional Animal Care and Use Committee. At the age of 7.5 weeks the rats were anesthetized with inhalation of 2.5% isoflurane in 0.5 l/min O_2_ flow. We used two approaches to register blood pressure. To study effects of salt intake (Figure 1), DSI telemeters (#TA11 PA-C40, Data Sciences International, St. Paul, MN) were installed in femoral arteries and affixed in a subcutaneous pocket in the loin area. Animals in individual cages were placed on an RPC-1 Transceiver interfaced via PhysioTel controller to computer equipped with the Ponemah software package.

**Figure 2:**
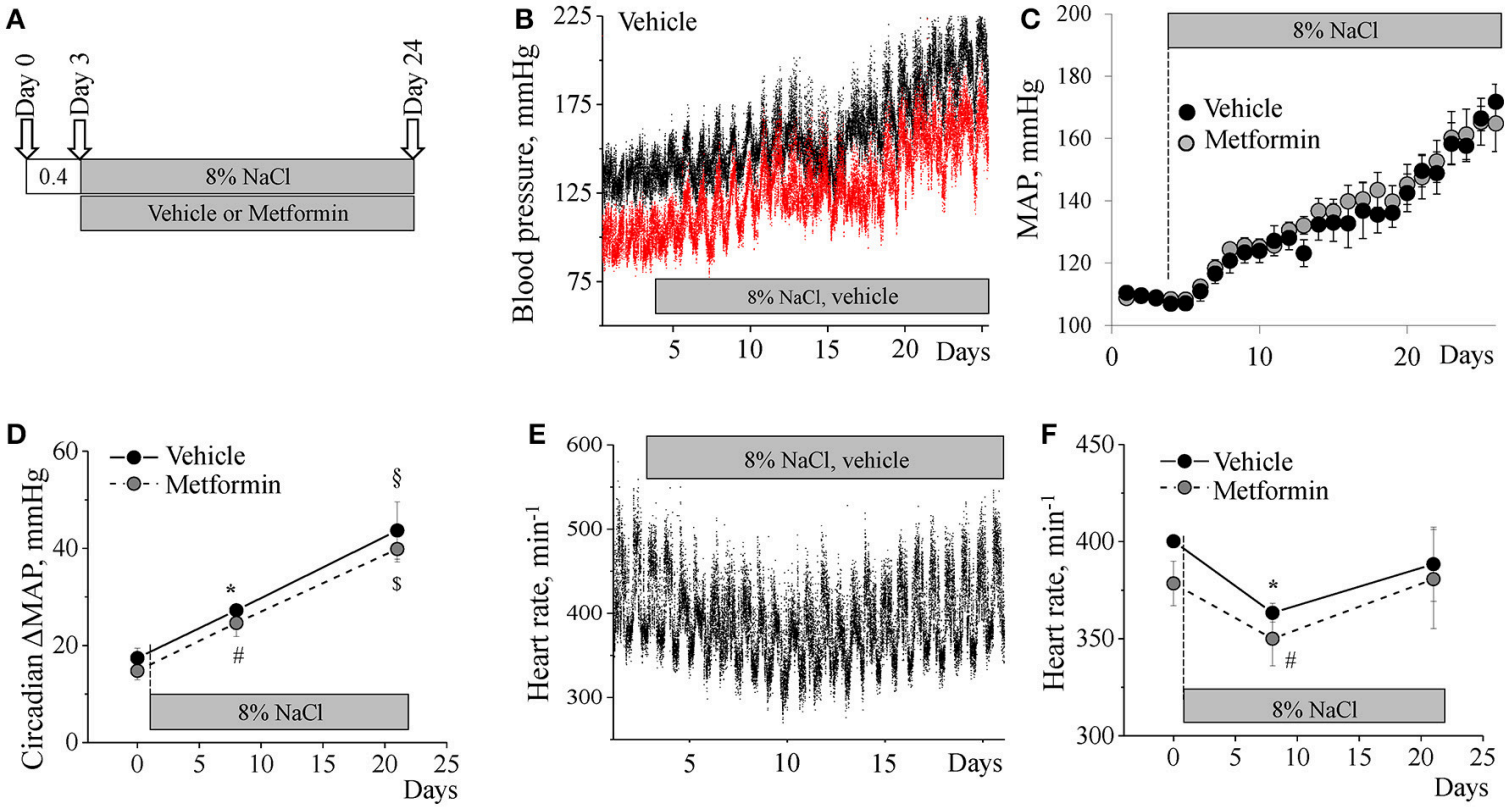
**Metformin does not alter the development of SS hypertension. (A)** Experimental protocol for BP recording. Continuous BP acquisition via catheters was performed in conscious Dahl SS rats placed on an 8% NaCl diet and subjected to an intravenous infusion of metformin (*n* = 9) or vehicle (*n* = 6). **(B)** Representative systolic (black) and diastolic (red) blood pressure recordings from a vehicle-treated animal. Data were averaged in 1 min intervals. **(C)** Mean arterial blood pressure (MAP) in the vehicle- and metformin-treated groups. **(D)** Metformin does not affect MAP circadian dipping increase during development of hypertension. **(E)** Representative heart rate trace acquired from a vehicle-treated animal. **(F)** Lack of effect of metformin on heart rate in experimental animals. ^*^,^#^*p* < 0.05 vs. 0.4% NaCl; ^§,*$*^*p* < 0.05 vs. 8 days on 8% NaCl.

**Figure 3:**
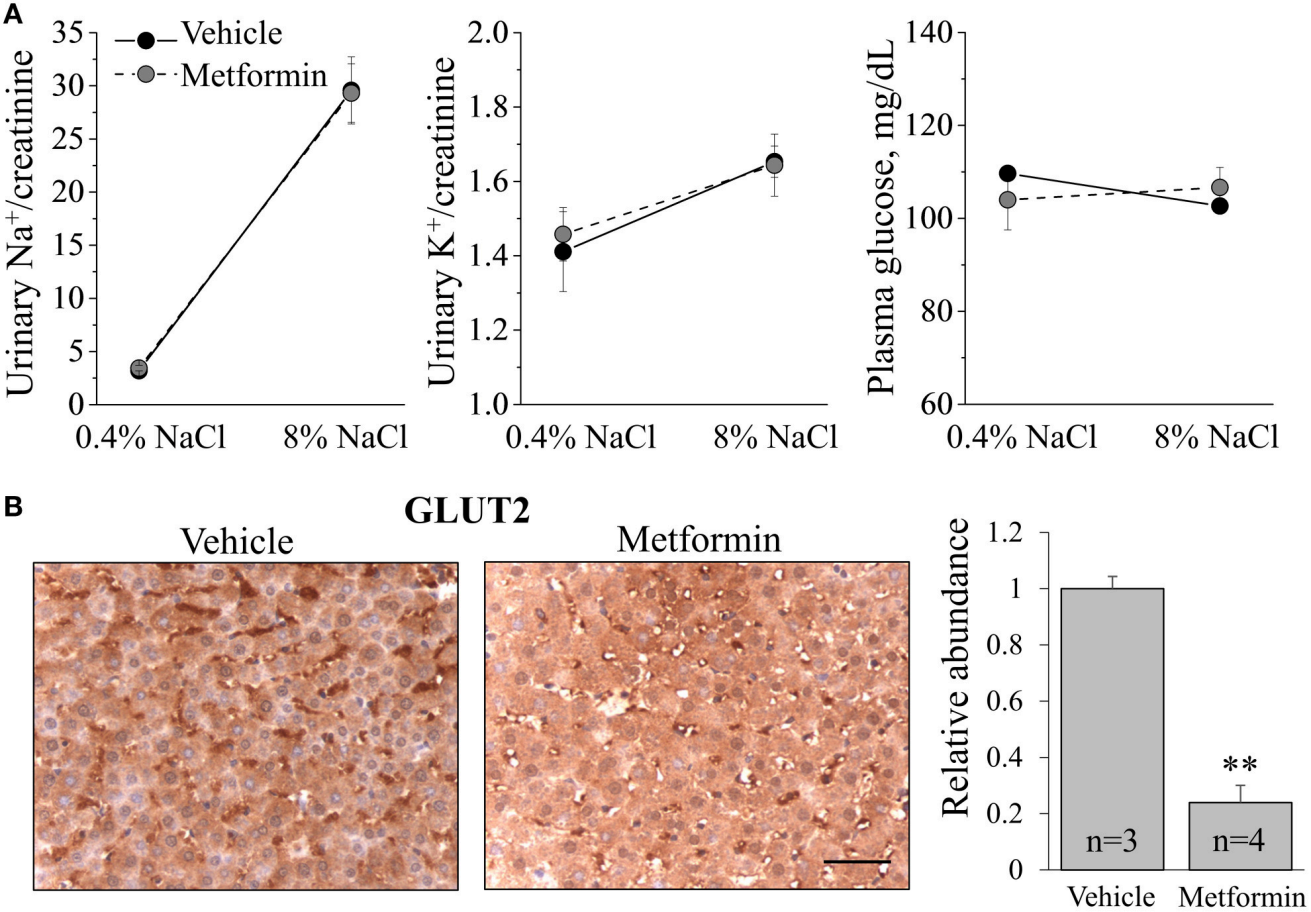
**Metformin treatment reduces Glut2 expression in the liver. (A)** Urinary sodium and potassium excretion and plasma glucose levels in vehicle- and metformin-treated rats. **(B)** Immunohistochemical staining demonstrates that metformin treatment decreases abundance of hepatic Glut2, a key glucose transporter involved in glucose release (40x). Scale bar = 50 μm. Shown on the right is summary graph for Glut2 relative abundance (^**^p < 0.01 relative to vehicle).

**Figure 4:**
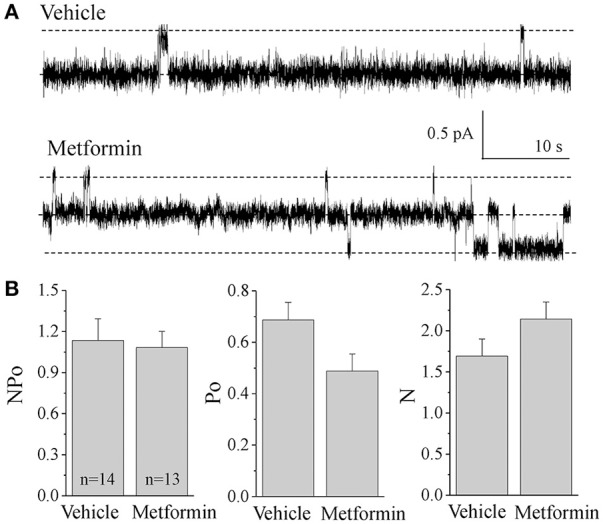
**ENaC activity in cortical collecting ducts of vehicle- and metformin-infused animals. (A)** Representative current traces for ENaC activity measured in cell attached patches from freshly isolated CCDs. Rats were fed with 8% salt diet and treated with either vehicle (*N* = 3) or metformin (*N* = 5). Holding potential was –40 mV. **(B)** Summary graph of observed ENaC activity (*NP*_*o*_), open probability (*P*_*o*_) of individual channels and number of channels (*N*) in the patches.

**Figure 5:**
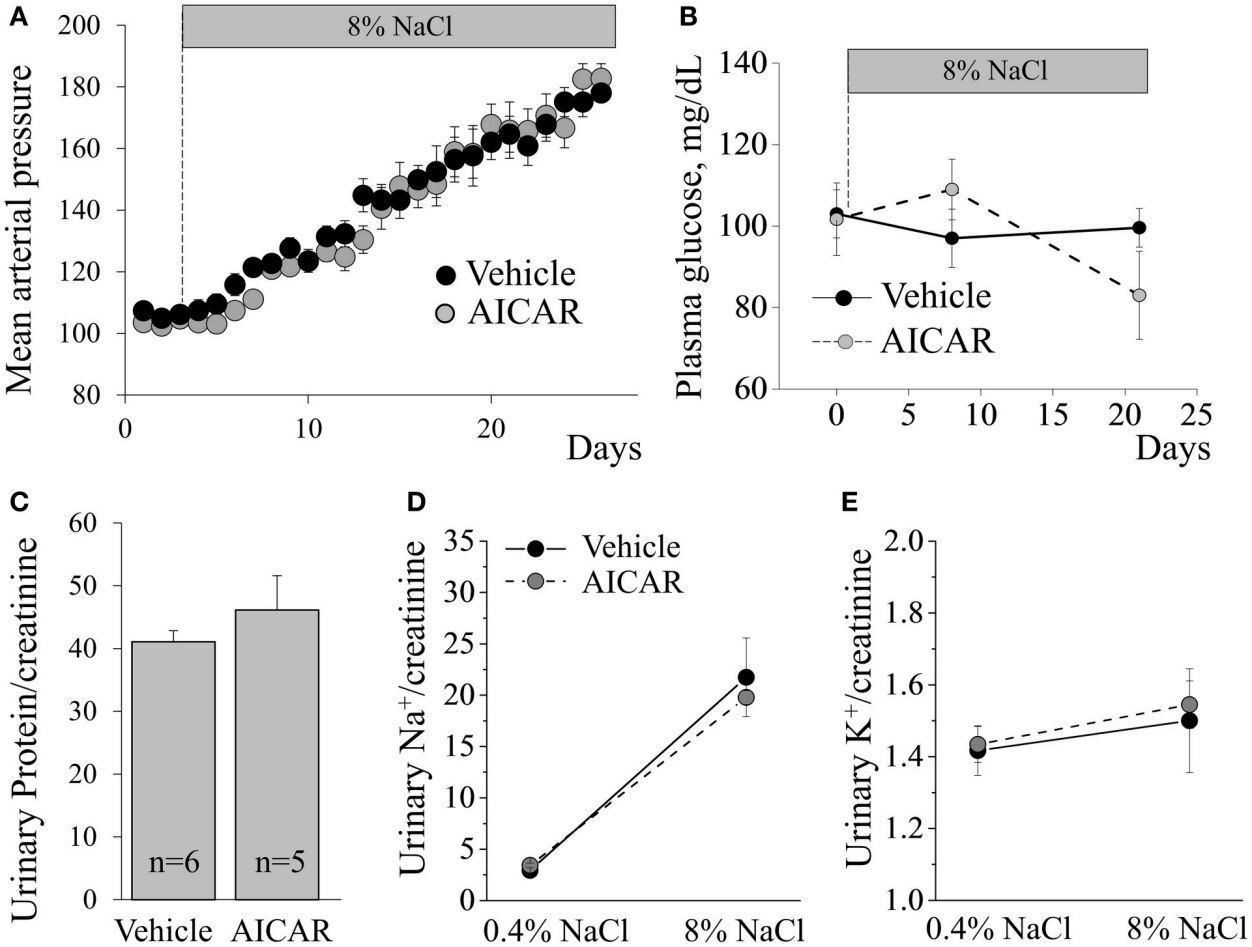
**AICAR does not alter development of SS hypertension. (A)** Blood pressure recordings collected from vehicle- or AICAR-treated Dahl SS rats during development of hypertension on an 8% NaCl diet (*n* = 6 and 5, respectively). Effect of AICAR treatment on the blood glucose level **(B)**, proteinuria **(C)**, sodium **(D)**, and potassium **(E)** excretion in Dahl SS rats fed an 8% diet. No significant differences between vehicle and AICAR-treated rats were found on any of the parameters tested.

**Figure 6:**
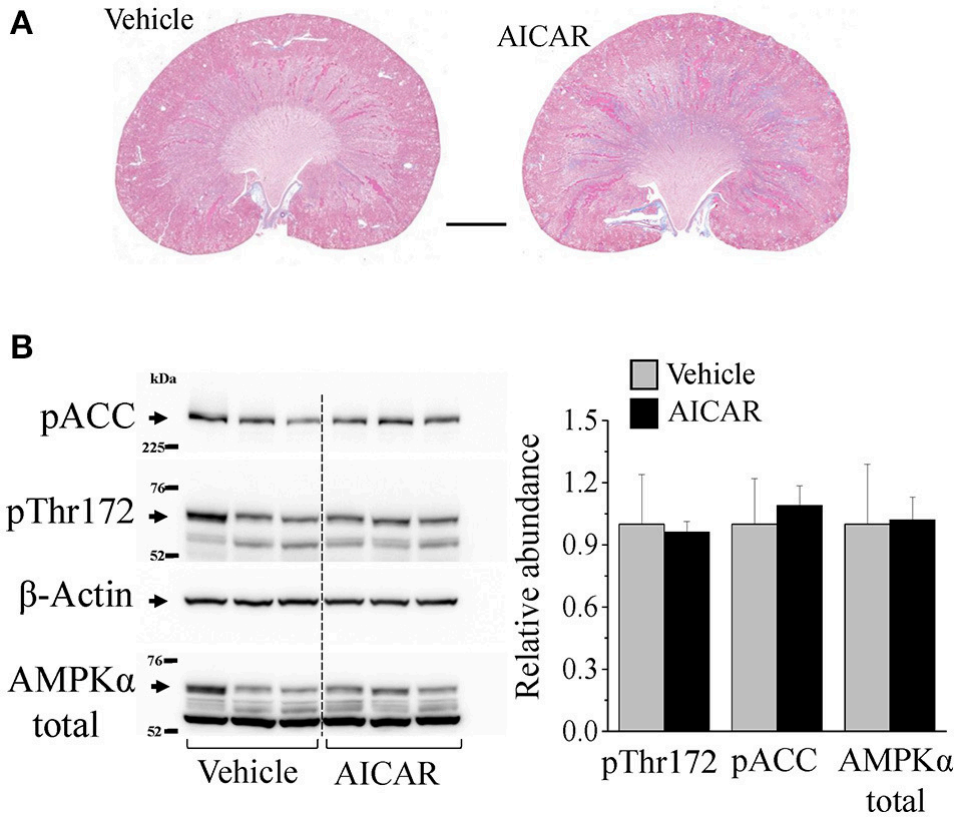
**AICAR treatment does not affect renal damage and cortical AMPK activity. (A)** Kidney morphology. Trichrome staining revealed that AICAR treatment did not preclude renal damage in SS hypertension. Scale bar = 2 mm. **(B)** Abundance of total AMPKα, phosphorylated Thr172-AMPKα and phosphorylated acetyl-CoA carboxylase (pACC), a canonical target of AMPK were studied in the renal cortex collected from the infused animals. No difference in AMPK activity was found between the groups.

For the experiments with drug infusion (Figures 2–**6**), catheters (#RPT080 Braintree, MA) were implanted in the femoral artery and vein, tunneled subcutaneously, and exteriorized at the back of the neck in a lightweight tethering spring (Moreno et al., 2007; Pavlov et al., 2013). Following recovery from anesthesia, all rats were placed into individual stainless steel cages that permit daily measurement of arterial blood pressure. The catheters were connected to pressure transducer (#041516504A, Argon Medical Devices, Plano, TX) via swivels (#375/D/22 Instech, Plymouth Meeting, PA) for arterial blood pressure acquisition and intravenous drug infusion with the syringe pump (10 ml/day). The rats were allowed to recover for at least 3 days following surgery.

### Experimental protocol

After the recovery period, blood pressure measurements were obtained continuously for the duration of the experiment. Plotted mean arterial pressure (MAP) data represent mean measurements from 9:00 a.m. to 12:00 p.m. as described previously (Pavlov et al., 2013). 20 mg/kg/day of AICAR or 200 mg/kg/day metformin (Tocris, Minneapolis, MN) administration was performed with continuous infusion at a rate of 6.9 μL/min through the venous catheter in saline, which was also used as a vehicle. After 3 days recovery when animals were fed a normal 0.4% NaCl diet, rats were switched to a 4 or 8% NaCl diet for 3 weeks (Dyets, Bethlehem, PA). Urine samples were collected during infusion experiments and Na^+^, K^+^, protein, and creatinine levels were analyzed as previously described (Pavlov et al., 2016). Blood was collected during the experiment with bleeds from the arterial catheters on conscious rats while resting, and plasma glucose level was then measured with a Contour strip glucometer (Bayer, Mishawaka, IN). At the end of the protocols, the kidneys of rats were perfused (6 ml/min) through the distal aorta with saline to clear them from blood, and then the renal tissues were collected for further analyses (Pavlov et al., 2015a).

### Western blot

Kidney cortical lysates were prepared as follows. Renal cortexes were excised rapidly and snap frozen in liquid nitrogen. Lysates were prepared using a Dounce homogenizer in lysis buffer [containing 20 mM Tris-HCl pH 7.4, 50 mM NaCl, 50 mM NaF, 5 mM Na-pyrophosphate, 250 mM sucrose, 1% Triton X-100, 1 mM DTT, 1 mM PMSF, and complete protease inhibitor cocktail (Roche)]. Homogenates were centrifuged at 16,000 × g for 30 min at 4°C, and the protein concentration in the supernatants was measured using the Bradford technique (Bio-Rad protein assay kit). Samples were separated by Nu-PAGE 4–12% gradient gels and electrically transferred to a polyvinylidene difluoride (PVDF) membrane (Bio-Rad). Membranes were cut horizontally at about 50 and 100 kDa to make three sections for probing. The top was probed for pACC. The middle was probed for pThr172-AMPKα. The bottom was probed for β-actin. The middle part was then stripped and reprobed for total AMPKα. We had similar results when we probed for AMPK-alpha first and then stripped and reprobed for pThr172-AMPKα (Li et al., 2015; Al-Bataineh et al., 2016). The membranes were blocked in 5% milk for 1 h and then incubated with primary antibodies in 5% BSA overnight at 4°C. After washes in TBS 0.05% Tween 20, the membrane was incubated with either goat anti-mouse or anti-rabbit secondary antibodies for 1 h at room temperature. Immunoreactive proteins were detected by the Versa-Doc imaging system (Bio-Rad). Quantification of Western blot bands was performed by densitometry using Quantity One software (Bio-Rad).

### Antibodies

Antibodies directed against AMPK-α, AMPK-α-phospho-Thr172, and ACC1-phospho-Ser79 (acetyl coenzyme A) were all obtained from Cell Signaling Technology (#2532, 2531, and 3661, Danvers, MA) and applied in dilution 1:1,000 as previously described (Li et al., 2015; Al-Bataineh et al., 2016). Anti-β-actin antibody was obtained from Sigma (A1978, St. Louis, MO; Pastor-Soler et al., 2015).

### Histochemistry

Formalin-fixed kidney sections were cut at 4 μm slices, dried and deparaffinized for subsequent streptavidin-biotin immunohistochemistry. After deparaffinization, the slides were treated with a citrate buffer (pH 6) for a total of 35 min. The slides were blocked with a peroxidase block (DAKO, Carpinteria, CA, USA), Avidin Block (Vector Labs, Burlingame, CA, USA), Biotin Block (Vector Labs), and serum-free Protein Block (DAKO). For kidney damage analysis, the tissue was stained with Masson's trichrome. For Glut2 staining, liver sections were embedded in one paraffin block for tissue microarrays and slices were incubated 1:200 overnight with anti-Glut2 antibodies (#sc-9117, Santa Crus Biotech, Dallas, TX). Secondary detection was performed with Goat anti-Rabbit biotinylated IgG (Biocare, Concord, CA, USA) followed by Streptavidin HRP (Biocare) and visualized with Diaminobenzadine (DAB, DAKO). ImageJ software was used to assess intensity of Glut2 staining in hepatocytes. Average signal levels in 10 areas in each liver were measured, and background (counterstained samples treated with secondary antibodies) was subtracted.

### Electrophysiology

Patch-clamp electrophysiology was used to assess ENaC activity in isolated, split-open rat cortical collecting duct (CCD). CCDs were isolated from Dahl SS rats as described previously (Pavlov et al., 2013, 2015b). Kidneys were cut into thin slices (< 1 mm) then placed into ice-cold physiologic saline solution (pH 7.4). Collecting ducts were mechanically isolated from these slices by micro-dissection using forceps under a stereomicroscope. CCDs were split open with sharpened micropipettes controlled with micromanipulators to gain access to the apical membrane.

Single-channel data were acquired using Axopatch 200B or 700B amplifiers (Molecular Devices, Sunnyvale, CA, USA) interfaced via a Digidata 1440 to a PC running pClamp suite software (Molecular Devices). When signals were acquired with an Axopatch 200B amplifier, currents were filtered with an 8-pole, low pass Bessel filter LPF-8 (Warner Inst., Hamden, CT) at 0.2 kHz. The pipette was pulled with a horizontal puller (Sutter P-97; Sutter Inst., Novato, CA). The resistance of the pipette in the corresponding bath medium was 7–12 MΩ. Typical bath solution was (in mM): 150 NaCl, 1 CaCl_2_, 2 MgCl_2_, 10 HEPES (pH 7.4). Pipette solution for the cell-attached configuration was (in mM): 140 LiCl, 2 MgCl_2_ and 10 HEPES (pH 7.4). Gap-free single channel current data from gigaohm seals in principal cells were acquired and subsequently analyzed with Clampfit 10.2 software (Molecular Devices). *NP*_*o*_, the product of the number of channels (*N*) and the open probability (*P*_*o*_), was used to measure the channel activity within a patch. Single-channel unitary current (*i*) was determined from the best-fit Gaussian distribution of amplitude histograms. Channel activity was analyzed as *NP*_*o*_ = *I*/*i*, where *I* is mean total current in a patch and *i* is the unitary current at this voltage. Where appropriate, *P*_*o*_ was calculated by normalizing *NP*_*o*_ for the total number of estimated channels in the patch.

### Statistics

All summarized data are reported as mean ± *SEM*. Data were compared using one-way ANOVA or Wilcoxon signed-rank test in case of dependent values; *P* < 0.05 was considered significant.

## Results

### Salt-induced blood pressure elevation is associated with AMPK activation

Dahl SS rats are known to develop hypertension when fed a high-salt diet (Rapp, 1982). First, we confirmed this effect using the DSI telemetry to monitor blood pressure in the rats fed 0.4, 4, or 8% NaCl diets between 8 and 11 weeks of age. The experiment revealed that during the 3-week period the 0.4% diet does not alter blood pressure, whereas the 4% diet raised MAP, and the 8% diet augmented the development of hypertension (Figure 1A). High NaCl diets increased sodium excretion proportionally to sodium content in the chow whereas potassium excretion remained stable, suggesting that food intake was similar on all diets (Figures 1B,C). While 4% diet represents a widely used dietary challenge, 8% diet had more profound and faster effects on blood pressure. Therefore, we used 8% in our subsequent experiments. At the end of the experiments, kidney samples were collected for the assessment of protein expression. No significant differences in terminal total body weights were found: average mass was 325.7 ± 9.7 (0.4% NaCl) vs. 308.5 ± 1.3 g (8% NaCl).

Western blot analysis in kidney cortex samples with antibodies that probe both α_1_ and α_2_ AMPK forms (559 and 552 amino acids length, respectively) detect four major immunoreactive bands (Figure 1D). Earlier, it was shown in rat liver tissue that the AMPKα signal might migrate as doublets. The authors also demonstrated that inactivation of AMPKα resulted in a shift in the migration of the upper band of the doublet to that of the lower band indicating that the two bands represent different phosphorylation states of the same enzyme and that activation is accompanied by a shift to higher bands (Carling et al., 1994). Our further investigations revealed similar forms of AMPKα which degrade in the renal tissues of AMPKβ1 knockout mice (Christensen et al., 2016). In the current study, high-salt diet causes increased activation of AMPK pathway in the renal cortex. 8% NaCl diet significantly increased both total AMPKα subunit abundance and phosphorylation of the Thr172 residue in the “activation loop” of AMPKα subunit (Stein et al., 2000). Phosphorylation of acetyl-CoA carboxylase (ACC), an immediate downstream effector and canonical target of AMPK, was also increased suggesting that high salt intake or high BP increases cortical AMPK activity (Figures 1D,E).

### Metformin treatment does not affect development of salt-sensitive hypertension

We tested whether the AMPK activator metformin affects blood pressure elevation in Dahl SS rats. After catheter implantation, recovery period and an initial 3 day blood pressure acquisition on a 0.4% NaCl diet, an 8% salt supplement along with vehicle or metformin infusion were instituted for an additional 21 day period (Figure 2A).

This high-salt challenge induced development of hypertension within the experimental period. Figure 2B shows a representative increase of systolic (black) and diastolic (red) blood pressures in a Dahl SS rat infused with vehicle. The BP elevated in two phases. The first stage was characterized by a sharp increase occurring over 1–8 days after the change of diet with an increase in MAP by ~21 mmHg. Then BP rose slowly by ~8 mmHg over 8–14 days from the switch in diet. Finally, during the 3rd week after switching to a high-salt diet there was a second acute phase when MAP rapidly rose up to 171.8 ± 5.6 mmHg (Figure 2C, black dots).

Development of hypertension was also accompanied with an increasing circadian BP dipping response: the difference between day/night MAP on a low-salt diet was 17.4 ± 2.1 mmHg whereas on a high-salt diet this value rose to 27.2 ± 0.8 mmHg on day 8 and 43.6 ± 5.9 mmHg by the end of experiment (Figure 2D). We also observed a temporary significant drop in heart rate after switching the diet, which recovered by day 21 (Figures 2E,F).

Metformin treatment in the high-sodium treated animals had no influence on the pattern of hemodynamic changes: neither MAP, circadian dipping, nor heart rate differed between vehicle- and metformin-treated groups (Figures 2C,D,F). During the experiment, sodium and potassium urinary excretion, plasma glucose level (Figure 3A) as well as total body weight at the end of the experiment (303.8 ± 12.5 vs. 309.6 ± 13.8 g) did not differ between the groups.

### Metformin decreases abundance of glut2 transporter in the liver

Metformin is well-known to modulate cellular metabolism in different tissues. Glut2 is a glucose transporter that mediates diffusive glucose transport through the plasma membrane in a variety of tissues (Thorens, 2015). To confirm that the infusion of metformin reached its targets and had functional effects, we assessed Glut2 expression in liver tissue collected after vehicle and metformin treatment. Immunohistochemistry assays revealed that metformin dramatically decreased the abundance of Glut2 in hepatocytes (Figure 3B). Therefore, 200 mg/kg/day intravenous metformin infusion had functional consequences in this rat model, altering Glut2 expression and presumably liver metabolic function.

### Lack of effect of metformin on ENaC activity

We recently demonstrated that high ENaC activity contributes to the development of hypertension in Dahl SS rats (Pavlov et al., 2013). We and others have shown that the AMPK pathway regulates ENaC activity in cultured cells and *in vivo* (Carattino et al., 2005; Almaça et al., 2009; Myerburg et al., 2010), although it is unclear whether this mechanism is functional in the Dahl SS rats. Therefore, here we studied whether the AMPK activator metformin has an effect on the activity of ENaC in the experiments described above in this important animal model of hypertension. CCDs were manually isolated immediately after organ collection, and patch-clamp analysis was performed on split-open tubules to assess channel activity. Figure 4A demonstrates representative ENaC current traces recorded from the apical membrane of vehicle- and metformin-treated Dahl SS rats. The analysis did not reveal any significant differences in total ENaC activity (*NP*_*o*_), open probability of individual channels (*P*_*o*_) or the number of channels (*N*) observed in patches (Figure 4B). Therefore, in this rat model of hypertension, metformin treatment does not decrease ENaC activity.

### AICAR treatment does not affect development of salt-sensitive hypertension

Considering the lack of effect of metformin on blood pressure elevation in Dahl SS rats, we tested whether another AMPK-activating pharmacological agent, AICAR, attenuates high blood pressure. Similarly, we found that 20 mg/kg/day continuous AICAR administration had no effect on the development of hypertension (Figure 5A). Analysis of the plasma samples collected via arterial catheters revealed that both vehicle- and AICAR-infused animals were euglycemic during the experiment (Figure 5B). By the end of the study, both groups demonstrated significant proteinuria, and neither sodium, potassium, total urine (73 ± 1.2 vs. 72.5 ± 8.4 ml/day) excretion, nor total body weight (288.4 ± 14.7 vs. 306 ± 7.3 g) differed between the two groups (Figures 5C–E). Histological analysis revealed massive abundance of protein casts, indicating kidney damage in the renal tissues in both groups (Figure 6A). The abundances of p-ACC, pThr172-AMPKα and total AMPKα, markers of AMPK pathway activity, in the cortex of AICAR-treated animals were similar to those of the control group as tested by Western blotting (Figure 6B).

## Discussion

AMPK activators have proven themselves to be useful drugs for metabolic disorders, including those associated with high blood pressure. Recently, Lazo-Fernández and colleagues determined that systemic knockout of α1 combined with kidney epithelium-specific deletion of α2 AMPK subunits led to a water and salt wasting phenotype in mice. The authors also suggested that activity of NKCC2, which serves as a substrate for phosphorylation by AMPK, might be reduced with AMPK targeting (Lazo-Fernández et al., 2017). Another example of the effect of AMPK on the renal concentrating ability in normal animals comes from experiments on kidney slices isolated from Sprague Dawley rats where AICAR treatment decreased apical membrane localization of AQP2, which could have a diluting effect on urine (Al-Bataineh et al., 2016). The current study was aimed at investigating the prospect of targeting AMPK for the treatment of salt-sensitive hypertension. Earlier, it was shown with a precise servo-control of body fluid volume that fluid retention is required to trigger the rise of pressure in Dahl SS rats (Greene et al., 1990).

Four and even 8% sodium chloride diets are commonly used; for instance, it was demonstrated that Dahl SS rats on an 8% NaCl diet had similar plasma sodium and protein levels compared to those on a 0.3% salt diet (and salt-resistant Dahl R rats on 8% diet), whereas their urine flow and sodium excretion rates were significantly higher as measured in pressure-natriuretic response studies (Roman and Kaldunski, 1991; Kakizoe et al., 2009). We have also shown that Dahl SS rats maintain stable plasma sodium and potassium levels when switched to 8% NaCl (Pavlov et al., 2013). We conclude that the rats could tolerate even such a high salt load as 8% NaCl chow when they have unlimited access to fresh water.

In the current study, salt-induced hypertension developed in two phases, early and late, which is typical for Dahl SS rats (Moreno et al., 2011; Evans et al., 2015). We observed an elevation in MAP and a progressive increase in day/night blood pressure dipping in all animals fed a high-salt diet, consistent with earlier findings (Mori et al., 2008; Cowley et al., 2016). In hypertensive and normotensive humans, nocturnal dipping is usually defined as a >10% decline in BP (Henskens et al., 2008) and has an important diagnostic significance: it has been shown that impaired BP dipping is associated with increased risk of cardiovascular events and target organ damage (Ohkubo et al., 2002; De La Sierra et al., 2009). In subjects from African descent, sodium excretory renal function during daytime was found to be a significant determinant of nocturnal BP and dipping (Bankir et al., 2008), whereas salt-resistant normotensive individuals' BP was not blunted on standard and high-sodium diets (Brian et al., 2017). Also, metabolic syndrome is recognized as a predictor of non-dipping hypertension (Tartan et al., 2006), and we expected that targeting AMPK activity might have an effect on circadian dipping in Dahl SS rats. However, in the current study blood pressure elevation was not accompanied by impaired dipping in either vehicle- or drug-treated animals. High-salt diet also substantially increased the activity of AMPK in the kidney cortex as revealed by Western blotting. We further tested whether treatment with the AMPK activators metformin and AICAR (given via intravenous infusion) affects the development of hypertension.

We showed that metformin infusion at a dose of 200 mg/kg/day reduced Glut2 abundance in hepatocytes, which demonstrated effective delivery and the targeting of liver function by the drug. Glut2 is a bidirectional glucose transporter present in liver, kidney, intestine, and pancreas, which facilitates glucose transit from the interstitium to the cell and vice versa. Studies have shown that Glut2 is overexpressed under diabetic conditions due to the increased glycolysis associated with the enhanced glucose transport (Zhang et al., 2013). Over-expression of Glut2 in hepatic tissue was found in high-fat and fructose-induced type-2 diabetic animals. Anti-diabetic treatment reduced Glut2 expression in high-fat and fructose-induced type-2 rats (Narasimhan et al., 2015).

However, we found a lack of effect of metformin on hypertension development, ENaC activity, and heart rate changes in this animal model. Similarly, earlier studies by Zhang and colleagues have shown that metformin did not affect development of hypertension in Dahl SS rats fed a 3% NaCl diet (Zhang et al., 1994). On the other hand, Yu et al. recently reported that chronic treatment with caffeine, which activated AMPK, increased natriuresis and precluded 8% NaCl salt-induced hypertension. Also, these authors suggested that in cell culture studies this effect of caffeine might be mediated by downregulation of ENaC via AMPK activation, as the caffeine effect was blocked by the putative AMPK inhibitor compound C (Yu et al., 2016). However, these results should be interpreted with caution because compound C is known to have significant off-target effects on a number of other kinases and signaling pathways (Bain et al., 2007). We studied the effect of chronic metformin administration on ENaC activity in the freshly isolated CCD segments of these high-sodium fed animals and did not detect any significant difference in ENaC function as compared to vehicle-treated animals. The lack of effect in our experiments may well be due to our finding that AMPK was already strongly activated following the institution of a high-salt (8% NaCl) diet (Figure 1), a finding that is consistent with earlier work in rats fed a high-salt diet (Fraser et al., 2005). Similarly, no further AMPK activation by AICAR infusion, as compared to vehicle, was found in our studies using Dahl SS rats fed a high-salt diet (Figure 6). It is therefore conceivable that any potential effect of AMPK activation on the hemodynamic parameters we measured was already maximized even in vehicle-treated animals during this exposure period. It would thus be interesting to examine the potential role of AMPK modulation in other hypertension models where AMPK may not be already activated.

Our study and the investigation by Zhang et al. used metformin at the following doses: 200 mg/kg/day intravenously and a daily dose of 300–350 mg/kg in drinking water, respectively. Metformin is usually used in rats in the 100–300 mg/kg/day range orally or intravenously (Santuré et al., 2000; Choi et al., 2008). In patients with type-2 diabetes, the oral form of metformin is usually given at total doses of 500–2,000 mg daily (Stumvoll et al., 1995; Basu et al., 2008; Takahara et al., 2012). Interestingly, Santuré and colleagues found that 3 weeks of treatment with metformin improves the glucose disposal in SHRs but has no blood pressure-lowering effect and no influence on vascular responses to insulin. In another study, 25–200 μg/day intracerebroventricular administration of metformin to SHRs attenuated 8% NaCl diet-induced hypertension; 100–200 mg/kg doses were highly neurotoxic as demonstrated by a histological evaluation post-mortem (Petersen et al., 2001). Together, these considerations and findings suggest that the selection of experimental model, route of administration, and dosage may all be critical for studying the effects of AMPK activators on blood pressure.

The renal effects of AMPK activators have been characterized in less detail than in such systems as skeletal and cardiac muscle or gastrointestinal tract. In our study, we have not observed effects of AICAR infusion on renal salt handling during BP elevation, although high-salt intake itself activated the AMPK pathway even without AICAR treatment, as discussed above. Another factor that could contribute to the observed lack of effect of the AMPK activators on BP might be that the dose was insufficient to target the kidneys. However, 200 mg/kg/day metformin did induce hepatic effects, which suggests the physiological efficacy of this infusion. AICAR is often used at high doses: antidiabetic effects are exhibited at doses of ~1 g/kg/day intravenously in humans (Boon et al., 2008) and rodents (Minokoshi et al., 2002). The safety and kinetics study in healthy men demonstrated that AICAR is poorly bioavailable (< 5%) when administered orally in solution; after intravenous injection the elimination phase half-time (t_1/2_β) was 1.4 h (Dixon et al., 1991). Our much lower intravenous dose of 20 mg/kg/day administration of AICAR did not exert any effects.

We conclude that the observed inability of metformin and AICAR at moderate and low doses to modulate blood pressure and renal salt handling in the Dahl SS rat challenged with a high salt diet warns against direct translation of these drugs toward the treatment of salt-sensitive hypertension. Further, studies are required to define an experimental approach and appropriate animal models for further testing the potential applicability of AMPK activators to this pathological condition.

## Ethics statement

This study was carried out in accordance with the recommendations of the Public Health Service (PHS) Policy on Humane Care and Use of Laboratory Animals. The protocol was approved by the Institutional Animal Care and Use Committee of Medical College of Wisconsin.

## Author contributions

TP drafted the manuscript and figures, performed patch clamp experiments, analyzed *in vivo* data, and collected samples. VL carried out and analyzed *in vivo* experiments. DI and OP participated in patch clamp experiments. HL and NP conducted western blot experiments. KH and AS developed study design and participated in data analysis and interpretation. All authors significantly contributed to writing of the text, figure preparation, and final editing.

### Conflict of interest statement

The authors declare that the research was conducted in the absence of any commercial or financial relationships that could be construed as a potential conflict of interest.
